# Hypothalamic Fatty Acids and Ketone Bodies Sensing and Role of FAT/CD36 in the Regulation of Food Intake

**DOI:** 10.3389/fphys.2019.01036

**Published:** 2019-08-14

**Authors:** Christelle Le Foll

**Affiliations:** Institute of Veterinary Physiology, Vetsuisse Faculty, University of Zurich, Zurich, Switzerland

**Keywords:** fatty acid, CD36, ketone bodies, neurons, astrocytes, hypothalamus

## Abstract

The obesity and type-2 diabetes epidemic is escalating and represents one of the costliest biomedical challenges confronting modern society. Moreover, the increasing consumption of high fat food is often correlated with an increase in body mass index. In people predisposed to be obese or already obese, the impaired ability of the brain to monitor and respond to alterations in fatty acid (FA) metabolism is increasingly recognized as playing a role in the pathophysiological development of these disorders. The brain senses and regulates metabolism using highly specialized nutrient-sensing neurons located mainly in the hypothalamus. The same neurons are able to detect variation in the extracellular levels of glucose, FA and ketone bodies as a way to monitor nutrient availability and to alter its own activity. In addition, glial cells such as astrocytes create major connections to neurons and form a tight relationship to closely regulate nutrient uptake and metabolism. This review will examine the different pathways by which neurons are able to detect free fatty acids (FFA) to alter its activity and how high fat diet (HFD)-astrocytes induced ketone bodies production interplays with neuronal FA sensing. The role of HFD-induced inflammation and how FA modulate the reward system will also be investigated here.

Over the past decade, the research on FA and how it can modulate neuronal signaling has grown substantially leading us to discover the role of cells that were under-studied until now, the astrocytes. This review will examine in detail how FA and its metabolites, such as ketone bodies, are able to alter astrocytes metabolism and neuronal activity to control food intake and energy homeostasis. Moreover, while FA can be uptaken directly from the blood stream and act on hypothalamic neurons through the FA transporter/receptor FAT/CD36, triglycerides/triacylglyceride (TG) are also found in the brain in lipoproteins. When TG are released by the lipoprotein lipase (LPL), subsequent FA can then alter neuronal activity. These FA, when in excess, are metabolized by astrocytes into ketone bodies to modulate neuronal activity. In addition to food intake and energy homeostasis, FA can also exert rewarding properties.

## Fatty Acids Brain Uptake and Transport

Fatty acid are first classified by their carbon (C) chain length: short-chain (<12 C), medium-length (between 12 and 18 C) and long-chain (>18 C). Then they are further classified by the number of double bonds: saturated (no double bonds), monounsaturated (one double bond) and polyunsaturated (>one double bond) (Table 1). The proportion of each FA absorbed and metabolized by the body depends on our food sources and dietary intake. While saturated FA have been associated with various deleterious effects on metabolism (Vessby, 2003) and cardio-vascular function (Mente et al., 2017), mono- and polyunsaturated FA present beneficial properties (Bellenger et al., 2019). Dietary fat is mainly composed of TG, cholesterols ester and phospholipids (Thomson et al., 1989). Briefly, once digested by enzymes in the mouth, stomach and small intestine, TG are broken down into smaller particles such as monoglycerides, FFA and cholesterol; these are then reorganized in micelles and uptaken by intestinal cells (Ramirez et al., 2001; Mu and Hoy, 2004) and released into chylomicrons in the blood stream to reach muscle, adipose tissue and the liver.

**TABLE 1:** Name and classification of the most common dietary fatty acids (FA).

**Name**	**Number of carbon: Number of double bonds**
**Saturated FA**	
*Octanoic acid*	C8:0
*Lauric acid*	C12:0
*Palmitic acid* (*PA*)	C16:0
*Stearic acid*	C18:0
*Arachidic acid*	C20:0
**Unsaturated FA**	
*Palmitoleic acid*	C16:1
*Oleic acid* (*OA*)	C18:1
*Linoleic acid* (*LA*)	C18:2
*Arachidonic acid* (*AA*)	C20:4
*Eicosapentaenoic acid* (*EPA*)	C20:5
*Docosahexaenoic acid* (*DHA*)	C22:6

To penetrate the brain, circulating FA need to cross the blood brain barrier (BBB). The BBB is a complex structure composed of endothelial cells that line the vessel wall, pericytes, astrocytes foot processes and a basal lamina (Engelhardt et al., 2014; Daneman and Prat, 2015). Together with neurons and microglia, the BBB forms the neurovascular unit with astrocytes providing a cellular link to adjacent neurons (Abbott et al., 2010; Banks, 2019). Endothelial cells of the BBB protect the brain against potentially dangerous substances through tight junctions. Additionally, most brains areas lack fenestrations, limiting paracellular passage (Brightman and Reese, 1969; Fenstermacher et al., 1988; Hawkins and Davis, 2005). To bypass the BBB, FA can passively diffuse through the BBB or are specifically transported at the membrane of brain capillaries (Hamilton, 1999).

Thus, FA can diffuse passively at the outer layer of the plasma membrane and this diffusion is dependent upon the lipophilic properties of the FA (Hamilton, 1999). Short-chain FA display the highest permeability while FA with a carbon chain over 12 C cannot directly penetrate the BBB because of their ionic charge (Levin, 1980). To diffuse rapidly across the plasma membrane, long-chain FA such as palmitic acid (PA), arachidonic acid (AA) and docosahexaenoic acid (DHA), which form a large proportion of the brain FA, need to be first transformed into a non-ionized form. This then allows a mechanism of “flip and flop,” which is independent of transport proteins (Hamilton et al., 2002, 2007; Kamp et al., 2003). In addition, the passage of these long chain FA is greatly facilitated when combined to albumin. In awake rats and using radioactive FA, Rapoport et al. (1997) have shown that when FA are first combined to albumin, they are rapidly uptaken and incorporated into membrane phospholipids. Similarly the carotid perfusion of ^14^C palmitic combined to albumin showed its rapid incorporation (less than 45 s) to the FA in the rat cerebral membrane phospholipids (Smith and Nagura, 2001). On the other hand, FA can use specific saturable transporters which have been located at the surface of the human and rodent endothelial cells (Mitchell and Hatch, 2011; Mitchell et al., 2011; Pelerin et al., 2014). These different transporters have been shown to be expressed in brain endothelial cells: FA transport protein (FATP) 1 and 4 (Fitscher et al., 1998; Ochiai et al., 2017), FAT/CD36 (Abumrad et al., 2005) and intracellular FA binding proteins 1-7 (FABP) (Zimmerman et al., 2001).

In human brain microvessel endothelial cells, FAT/CD36 performs the majority of the transport (Mitchell et al., 2011). A similar level of brain PUFA was shown in CD36 knockout mice vs. WT mice suggesting that CD36 is not the only mechanism maintaining brain PUFA levels in these KO mice (Song et al., 2010). This was further confirmed by Pelerin et al. (2014) who quantified the different transporter in the BBB of the rat cerebral cortex with FATP4 being the most abundant. FATPs are bifunctional proteins (uptake and acylation) and do not display any affinity for a specific type of FA (Schaffer and Lodish, 1994; Stahl et al., 1999). These FATPs are well-conserved across species and have two domains, the ATP binding domain and the FA binding domain (DiRusso et al., 2008). FATPs are coupled to a long-chain acyl-coA synthetase which transforms FA into FA acyl-CoA to be directly metabolized by the adjacent cells (Hall et al., 2003). To mediate FA uptake and metabolism, the binding of ATP is necessary and whether the change in ATP/AMP ratio affect the adjacent neuron and astrocyte activity remains to be determined.

FAT/CD36 binds saturated and unsaturated FA in the nanomolar concentration range (Baillie et al., 1996) and has been shown to have a higher affinity to long-chain FA (>18 C) at least in mice enterocytes (Drover et al., 2008). The exact mechanism by which it transports FA still remains to be elucidated. FABP1, 3, 5, and 6 are also expressed in other tissues than the brain, such as the liver and intestine, while FABP7 is specifically expressed in the rat brain (Veerkamp and Maatman, 1995; Glatz and van der Vusse, 1996). FABP5 is highly expressed by human and mice endothelial cells (Masouye et al., 1997; Pan et al., 2015). Using cell line and human brain FABPs constructs, long-chain unsaturated FA such as oleic acid (OA) and AA seem to present a higher affinity compared to PA (Zimmerman et al., 2001). When studying these transport processes in rodents, it is important to notice that their expression is variable depending on the metabolic status and the species. For example, rats fed with n-3 FA enriched diet had an increase in brain microvessels FABP7 compared to chow-fed rats and brain FA transporters were decreased in 9-week old rats compared to 3-week old rats (Pelerin et al., 2014).

While these transport mechanisms are important to brain areas with a BBB, circumventricular organs (CVO) which are located at the interface between the brain and the periphery present a highly fenestrated BBB. These sensory CVOs are: subfornical organ; organum vasculosum of the lamina terminalis; area postrema; median eminence. In the medio-basal hypothalamus, a crucial area in the control of food intake, the ARC also presents a “leaky” BBB due to the presence of tanycytes and fenestrated capillaries (Dehouck et al., 2014; Banks, 2019). In these regions, the lesser number of tight junctions between endothelial cells allows the penetration of molecules (Bennett et al., 2009). The uptake of FA in these regions has been scarcely studied (Banks et al., 1997) and one can assume that the presence of fenestrated endothelial cells facilitates the passage of FA.

## Brain FA Receptors and Modulation of Neuronal Activity

While during fasting, FFA levels are increased due to lipolysis, the elevation of FFA during HFD feeding stimulate glycolysis and lipogenesis. Metabolic sensing neurons located in the medio-basal hypothalamus monitor the variation of FFA, glucose and insulin levels along with other gut hormones to alter their activity and act on diverse peripheral mechanisms involved in the control of food intake. While short-term HFD feeding triggers food satiety, long-term HFD dysregulates these processes and food intake control is altered.

### Hypothalamic Nutrient Sensing and CD36-Mediated FA Neuronal Sensing

In the 1950s, Jean Mayer first hypothesized that neurons in the hypothalamus were able to sense a change in glucose oxidation as a mean of controlling feeding (Mayer, 1953). Later, Oomura et al. (1964) identified these neurons and named them glucosensing neurons. Indeed, the hypothalamus contains “metabolic sensing neurons” which monitor ambient levels of substrates such as glucose, FA (Le Foll et al., 2009b) and ketone bodies (Le Foll and Levin, 2016) but also of hormones such as leptin (Irani et al., 2008) and amylin (Davidowa et al., 2004). These neurons alter their membrane potential and activity in response to these stimuli resulting in the activation or release of a variety of substrate transporters, ion channels and enzymes (Anand et al., 1964; Oomura et al., 1964; Levin et al., 2006; Migrenne et al., 2006; Wang et al., 2006; Levin, 2007; Jo et al., 2009; Le Foll et al., 2009a,b, 2013, 2014, 2015a; Blouet and Schwartz, 2010; Chari et al., 2010) all these factors contribute to the control of feeding, thermogenesis and other facets of energy and glucose homeostasis (Mayer, 1953; Oomura et al., 1975; Levin et al., 2006; Migrenne et al., 2006; Wang et al., 2006; Levin, 2007; Jo et al., 2009; Blouet and Schwartz, 2010; Chari et al., 2010). These specialized neurons are clustered in brain areas such as the arcuate nucleus (ARC) (Muroya et al., 1999; Ibrahim et al., 2001; Wang et al., 2006; Le Foll et al., 2009b), ventromedial hypothalamic nucleus (VMN) (Song et al., 2001; Dunn-Meynell et al., 2002; Kang et al., 2004; Levin et al., 2004; Le Foll et al., 2009b), areas involved in energy and glucose homeostasis and neuroendocrine regulation. Many studies have demonstrated that FA sensing neurons are present in the VMN and ARC (Migrenne et al., 2006; Wang et al., 2006; Jo et al., 2009). Among the ARC neurons, the role of FA on POMC and NPY/AgRP neurons has been the most studied. POMC and NPY/AgRP neurons belong to the melanocortin system (Joly-Amado et al., 2014). POMC neurons release the catabolic neuropeptide, α-melanocyte stimulating hormone (αMSH) which acts on melanocortin 3 and 4 receptors (MCR3/4) to subsequently reduce food intake and increase energy expenditure via thermogenesis (Balthasar et al., 2005). AgRP serves as a potent endogenous antagonist at the MCR3/4. NPY/AgRP neurons synapse onto POMC neurons to provide inhibitory action via release of NPY onto NPY receptor type 2 (Y2) and gamma-aminobutyric acid (GABA), further supporting a tightly regulated neuronal network [for review see Morton et al. (2006)].

Studies by Jo et al. (2009) demonstrated that in mice, 40% of proopiomelanocortin (POMC) neurons in the ARC sense long-chain FA via β-oxidation and closure of the K_ATP_ channel. OA depolarizes and increases firing rate of POMC cells during patch-clamping and this depolarization is concentration dependent while octanoic acid, a short chain saturated FA does not induce the same effect in these same neurons. Out of these POMC FA sensing neurons, 60% were excited by glucose. Opposite to POMC, OA is unable to depolarize AgRP neurons (Jo et al., 2009) even though Flick et al. showed later on that PA increased NPY mRNA in hypothalamic cell line (Fick et al., 2011). When mice were fed chow or HFD for 2 weeks, HFD itself altered the neurons excitability but their response to OA was similar in both groups. Further Diano et al. (2011) observed that, in response to reactive oxygen species production (ROS), HFD-feeding decreased POMC firing which in turn increased food intake (Paeger et al., 2017). The inactivation in POMC neurons of the apoptosis–inducing factor, a factor that is involved in the mitochondrial respiratory chain, increased neurons FA utilization and ROS production increasing POMC firing rate (Timper et al., 2018). More recently, a study showed that a systemic infusion of PA increased POMC neurons and PC1/3 mRNA hypothalamic expression. The inhibition of PC1/3 promoted caloric intake and weight gain in an obesity resistant mouse model (Souza et al., 2016). Furthermore, PA also increased POMC transcript in human hypothalamic neurons and this effect was found with PA only (Tse and Belsham, 2018; Razolli et al., 2019). On the contrary, rats injected intracerebroventricularly with different FA, only OA and DHA (and not PA), were able to increase POMC mRNA expression. The use of an MCR4 antagonist completely reversed the anorectic effect of OA (Schwinkendorf et al., 2011). Taken together, these results suggest that POMC neurons may mediate the anorexigenic effects of OA. These studies demonstrate that FA can alter neuronal activity and that these effects are FA-specific.

While these above studies have used hypothalamic cell line and *in vivo* FA infusion, nanomolar concentrations of OA have been shown to either excite or inhibit 43 and 29% of VMN 3-week old rats dissociated primary neurons (Le Foll et al., 2009a). Pharmacological studies were then undertaken and Ca^2+^ imaging of dissociated primary neurons as a surrogate for changes in neuronal activity was measured. The exclusion one by one of each component of the classical β-oxidative FA intracellular metabolic pathway such as carnitine palmitoyltransferase 1 (CPT1), acetyl-CoA carboxylase (ACC) and K_ATP_ channel (Le Foll et al., 2009a) demonstrated that FAT/CD36 is the principal mediator of FA in modulating neuronal activity in the ventromedial hypothalamus (VMH = ARC + VMN) of mice and rats. Indeed only 20% of neuronal FA sensing is mediated by these classical intracellular pathways whereas 50% is mediated by FAT/CD36 (Le Foll et al., 2009a). FAT/CD36 is located throughout the brain, but its function in relation to food intake and energy homeostasis has mainly been studied in the medio-basal hypothalamus of rodents (Le Foll et al., 2009a, 2013, 2015a).

The depletion of FAT/CD36 in the ARC and VMN neurons using an AAV shRNA did not affect the glucosensing properties of 3-week old rats primary dissociated neurons, however, OA excitatory effect on VMH neurons was reduced by 50% and its inhibitory effect by 75% (Le Foll et al., 2013). On the other hand, the complete loss of CD36 in CD36KO mice affected the glucosensing properties by decreasing glucokinase, a critical regulator of glucosensing (Kang et al., 2004). CD36 KO mouse FA sensing neuronal response was also different than what was observed in rat primary neurons. Indeed, CD36 KO mice had fewer inhibitory FA sensing neurons, but the depletion of CD36 did not modify the excitatory properties of these neurons. These discrepancies could result from species difference and/or the depletion’s method. On one side CD36 was depleted postnatally using an AAV, on the other side CD36 whole-body KO resulted from a germ cell deletion where both plastic changes during development leading to compensatory mechanisms and overall altered energy and glucose homeostasis can occur. In addition, patients with depletion of the CD36 gene are often diagnosed with hyperinsulinism and are prone to metabolic syndrome underlining the importance of FAT/CD36 in the control of metabolism (Love-Gregory and Abumrad, 2011).

Similar to taste buds, it is hypothesized that CD36-mediated FA-induced neuronal activation increases [Ca^2+^]_i_ by recruiting Ca^2+^ from the endoplasmic reticulum pool which opens store-operated calcium channels (Abdoul-Azize et al., 2014); more experiments are needed, however, to validate this hypothesis. CD36-mediated FA sensing occurs specifically in ARC and VMN glucosensing neurons highlighting the idea that these metabolic sensing neurons integrate signals from metabolic substrates as well as hormones and neural inputs to regulate their activity (Levin et al., 2011).

While the important role for CD36 in mediating neuronal FA sensing in the VMH was established *in vitro*, its crucial function in the control of food intake, long-term energy and glucose homeostasis was further assessed. The depletion of CD36 using an AAV shRNA in the VMH of weanling Sprague-Dawley rats fed for 9 weeks 45% HFD increased leptin levels and subcutaneous fat and induced insulin-resistance without overall effect on food intake and body weight (Le Foll et al., 2013, 2015a). Since both obesity and type 2 diabetes have major comorbidities that make it imperative to understand the underlying mechanisms that control food intake and regulate energy and glucose homeostasis, the role of CD36 was further assessed in a rodent model of human obesity. Diet induced obese (DIO) and diet resistant (DR) rats are selectively bred to reproduce the polygenic alterations inherent to human obesity (Levin and Strack, 2008). These rats are selectively bred to produce DIO or to remain DR when fed a high energy (HE, 31% fat-24% sucrose as% of total energy source) diet. On low-fat chow diet, DIO rats are larger, but not fatter, than DR rats. When fed HE diet, DIO rats rapidly develop an insulin-resistance and become obese (Levin et al., 1997; Levin and Dunn-Meynell, 2000, 2002). The depletion of CD36 in the VMH of DIO rats fed 45% HFD increased their food intake and weight gain compared to AAV control DIO rats, while CD36- depleted DR rats gained less weight than DR AAV controls. VMH CD36 depletion increased inguinal fat pad weights and leptin levels in DIO and DR rats. Even though CD36-depleted DR rats became as obese as DIO AAV controls, only DIO CD36 depleted rats became insulin-resistant on a 45% HFD as shown by a glucose intolerance and by elevated insulin levels during the oral glucose tolerance test. In addition, liver TG were drastically increased in DIO rats injected with AAV CD36 shRNA. All together, these results demonstrate that VMH CD36- FA sensing neurons are of the most importance in the control of food intake and the regulation of energy and glucose homeostasis as well as fat deposition in DIO and DR rats (Le Foll et al., 2009b, 2015a).

In addition to FAT/CD36, other pathways can alter neuronal FA sensing. Indeed, manipulating brain FA oxidation itself can also alter food intake (Clegg et al., 2002; Obici et al., 2003; Pocai et al., 2006; Aja et al., 2007). Earlier studies have shown that the hypothalamic levels of long-chain fatty acyl-CoAs can be increased by enhanced esterification of circulating or central lipids (Obici et al., 2002; Lam et al., 2005) or by the local inhibition of lipid oxidation (Obici et al., 2003). These interventions result in a marked inhibition of liver gluconeogenesis and a decrease in food intake (Obici et al., 2002, 2003; Lam et al., 2005; Pocai et al., 2006). Further, the inhibition of FA synthase and the stimulation of FA mitochondrial metabolism through CPT1 decreased food intake and body weight in mice and rats (Aja et al., 2006, 2007). However, more recent studies suggest that the inhibition or stimulation of these FA metabolic pathways could affect astrocytes’ metabolism rather than neurons since astrocytes are the major source of brain FA oxidation (Edmond, 1992) (see section “Astrocyte-Neuronal Coupling and Regulation of Food Intake”).

### Role of Lipoprotein Lipase in Mediating Neuronal FA Sensing

While research has focused on the role of circulating FA coming from the periphery and crossing the BBB, lipoproteins are also found in the brain and these are produced within the central system and contribute to the brain lipid sensing (Cruciani-Guglielmacci and Magnan, 2017; Gao et al., 2017b). These lipoproteins are the main carrier for TG to be transported into the brain; lipoproteins are TG-enriched particles and are mainly found in the brain under the HDL form (Wang and Eckel, 2014). Several lipases are expressed in the brain and can locally hydrolyze these particles to release TG (Paradis et al., 2004). Similar to FFA and other nutrients, daily variation of TG level has been detected in the brain (Ruge et al., 2009) indicating that TG or their metabolic products are able to inform brain cells about the body nutrient status and participate in the control of energy homeostasis. LPL mRNA has been detected in neurons and astrocytes in the brain (Wang et al., 2011; Wang and Eckel, 2012) and LPL has been demonstrated to be the key enzyme that metabolize TG into FA and its activity is regulated by nutrients and hormones in a tissue-specific manner (Wang et al., 2011; Wang and Eckel, 2012). The depletion of LPL in the VMH induced body weight gain in mice associated to hyperinsulinemia and glucose intolerance as well as a decrease in locomotor activity (Laperrousaz et al., 2017). This suggests that the absence of LPL-mediated FA sensing in the brain may lead to deregulation of energy balance (Laperrousaz et al., 2017). It has also been shown that the depletion of LPL in the dorsal hippocampus increased body weight gain without affecting food intake in both mice and rats (Picard et al., 2014). This increase in body weight gain was associated with a decrease in locomotor activity and energy expenditure and as well as an increase in parasympathetic nervous activity (Picard et al., 2014). Last, the specific depletion of LPL in astrocytes reduced the accumulation of lipid droplets in these cells, increased body weight and induced a glucose intolerance in these high-fat diet fed-mice (Gao et al., 2017c). Together these studies seem to indicate that LPL is a major key enzyme regulating the hydrolysis of TG from lipoprotein thus providing FA which can then be detected by neurons and glial cells.

### G-Protein Receptors GPR120 and GPR40

Recently, other FA receptors have been discovered in the brain. These G-protein receptors, GPR120 and GPR40, have been found on neurons and microglia (Cintra et al., 2012; Dragano et al., 2017) in brain areas such as the ARC, ventral tegmental area (VTA), nucleus accumbens (NAc) and hippocampus (Auguste et al., 2016), although in much lower levels than in the intestine or the adipose tissue. These receptors are stimulated by long-chain FA including omega-3 FA such as eicosapentaenoic acid (EPA) and docosapentaenoic acid (DHA). Hirasawa et al. (2005) showed that the stimulation of GPR120 by long-chain FA activate ERK cascade which suggests interactions with the Gαq family of G proteins. Depletion studies of GPR120 with a lentivirus in mice did not alter body weight or food intake and no change in glucose tolerance was detected (Dragano et al., 2017). The injection of the GPR120 agonist decreased food intake acutely in chow-fed rats and suppressed the rewarding effect of high-fat high-sugar diet as well as anxiety-like behavior (Auguste et al., 2016). However, the activation of GPR40 with an agonist did not affect food intake (Auguste et al., 2016). It also appears that the activation of GPR120 produces anti-inflammatory effects at least in immortalized hypothalamic neurons (Wellhauser and Belsham, 2014).

## Astrocyte-Neuronal Coupling and Regulation of Food Intake

While the focus for the past decades has been on the role of metabolic sensing neurons, emerging data have shown that glial cells and particularly astrocytes can also play an important role in detecting and metabolizing FA to modulate energy homeostasis. Indeed, astrocytes, which first function provide metabolic support for neurons (Edmond, 1992; Yi et al., 2011), have been understudied until recently. Astrocyte foot processes are directly in contact with brain microvessels and thus are the first cells encountered by nutrients entering the brain (Abbott et al., 1992; Tsacopoulos and Magistretti, 1996). Astrocytes have several important metabolic functions (Pellerin and Magistretti, 1994; Magistretti and Pellerin, 1999; Perea et al., 2009, 2014; Araque et al., 2014) such as glycogen storage and lactate production to support neuronal metabolism, especially during enhanced neuronal activity (Pellerin et al., 1998; Pellerin and Magistretti, 2012). In addition, astrocytes can also release and transport neurotransmitters (Allen, 2014; Khakh and Sofroniew, 2015), regulate ion concentration and regulate blood flow (MacVicar and Newman, 2015) and act as glucose sensors (Garcia-Caceres et al., 2016).

### Production of Ketones by Astrocytes and Role of Tanycytes

During fasting period (see review Cahill, 2006), exogenous glucose levels decrease, inducing liver glycogenolysis and adipose tissue lipolysis (DeFronzo, 2004; Moore et al., 2012; Sharabi et al., 2015). Once glycogen stores are depleted, the liver first generates glucose through gluconeogenesis to fuel other tissues (Zhang et al., 2018). If the fasting condition persists, ketone bodies (β-hydroxybutyrate (βOHB) and acetoacetate) are generated by the liver from FA through mitochondrial β-oxidation and ketogenesis (Rui, 2014). Ketone bodies can also be produced by the intestine and stimulate local visceral afferents to regulate food intake (Karimian Azari et al., 2013; Azari et al., 2014). These ketones bodies, as well as FA, can be transported into the brain to serve as an alternate energy source during fasting (Rui, 2014). Many studies have assessed the role of ketone bodies on food intake by either infusing ketones (Park et al., 2011) or using ketogenic diets (Johnston et al., 2006; Dashti et al., 2007; Park et al., 2011). Ketogenic diets which are rich in fat and low in carbohydrates, have been of great interest recently. Briefly, dietary TG are digested by gastrointestinal lipase to generate FA, which are then metabolized by the liver through β-oxidation and transformed into ketone bodies due to the absence of carbohydrate (Schonfeld and Wojtczak, 2016). FA and ketone bodies are then transported across the BBB where they can be used as an energy source. The generation of ketone bodies provides astrocytes and neurons with energy sources that can be more efficiently used than glucose (Zhang et al., 2013). Further β-hydroxybutyrate has been shown to inhibit astrocyte glucose consumption in mouse astrocyte culture and consequently increase glucose neuronal availability (Valdebenito et al., 2016). Carneiro et al. (Carneiro et al., 2016) has shown that infusion of ketone bodies though the carotid artery in direction of the brain without elevating circulating blood ketone bodies transiently increases food intake and alter energy homeostasis. This paradigm reproduces the effects of ketones during fasting condition, confirming that ketone bodies levels can be detected by the brain to modify peripheral energy homeostasis. However, none of these studies have assessed if these ketone bodies can be produced by the brain *per se* when FA are in excess during HFD intake.

Hence, when energy intake is abundant and particularly rich in lipids, hypothalamic astrocytes can then produce ketone bodies from FA (Blazquez et al., 1999; Guzman and Blazquez, 2004; Escartin et al., 2007; Le Foll et al., 2014). Many studies have shown that astrocytes are the major brain site of FA oxidation and the only possible source of ketone body production in the brain (Edmond et al., 1987, 1998; Blazquez et al., 1999). During fasting or when blood glucose levels are low, FFA levels are elevated and are metabolized by astrocytes (Edmond et al., 1987) they then enter the mitochondria and undergo β-oxidation to provide ATP to adjacent neurons (Edmond et al., 1987; Pocai et al., 2006). During HFD feeding, i.e., when energy intake and FA are in excess, hypothalamic astrocytes can also produce ketone bodies (Edmond et al., 1987; Blazquez et al., 1999; Guzman and Blazquez, 2004; Le Foll et al., 2014). 3-hydroxy-3-methylglutaryl-CoA (HMG-CoA) synthase and HMG-CoA lyase are the rate- limiting enzyme involved in this process (Hegardt, 1995, 1998).

**FIGURE 1:**
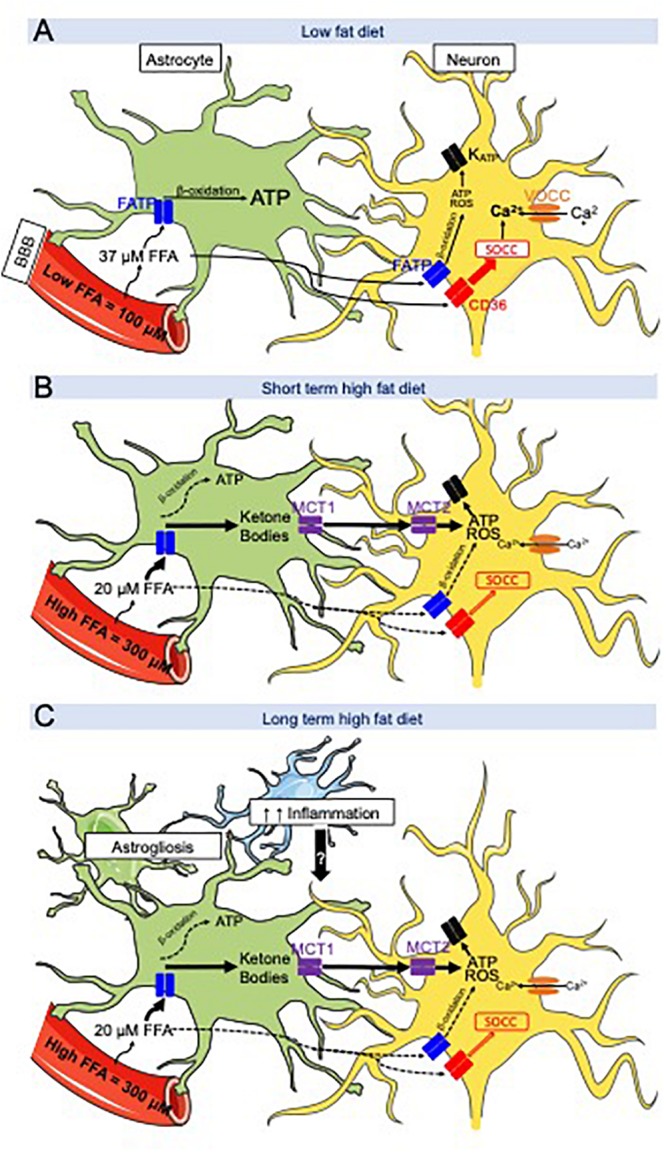
Hypothetical working model between CD36-mediated neuronal fatty acid (FA) sensing and astrocyte-produced ketone bodies during low fat diet (LFD) and high fat diet (HFD) intake. **(A)** LFD intake- FA crossing the blood across the blood-brain barrier (BBB) are taken up and oxidized by astrocytes to produce ATP while FA bind to FAT/CD36 located on metabolic sensing neurons in the ventromedial hypothalamus (VMH = VMN + ARC). Their excitation or inhibition activates store-operated Ca^2+^ channels (SOCC) which depolarize or hyperpolarize the neuron. **(B)** After a short-term HFD intake, brain extracellular FA levels are decreased compared to LFD intake due to an increase in FA flux into VMH astrocytes to generate ketone bodies. Ketone bodies are exported via MCT1 and taken up by neurons via MCT2. Ketone bodies are utilized by the neurons and produce ATP and ROS in excess. ATP and ROS are hypothesized to override CD36-mediated FA sensing which activate or inhibit the K_ATP_ channel. **(C)** Long term HFD intake induces as astrogliosis and microglial inflammation. These cytokines might interfere with the astrocyte-neuronal communication leading to disturbances in the control of food intake promoting continued hyperphagia on HFD. Adapted from Le Foll and Levin (2016).

During low-fat diet (LFD; 13.5% fat as percent of total energy intake) and after a 24 h fast, hypothalamic ketone bodies levels are ∼20 μM, whereas the FFA levels are ∼37 μM while their serum levels are ∼350 and ∼300 μM respectively (Le Foll et al., 2014; Figure 1A). However, when lean rats are fed a 60% HFD for several days on restricted intake schedule (3 h per day of feeding and 21 h of fasting), VMH FFA levels reach a concentration of ∼ 22.5 μM, which is lower than what was measured during LFD intake (Le Foll et al., 2014). On 60% HFD and using the restricted schedule paradigm, VMH ketone levels spike to ∼100 μM 1 to 2 h after the onset of feeding (Le Foll et al., 2014). To explain this difference in VMH FFA levels between LFD and HFD, we could hypothesize that during HFD intake, FA are uptaken at a greater rate by astrocytes which then metabolize them into ketone bodies. The monocarboxylate transporter 1 (MCT1) allows for the export of ketone bodies out of astrocytes, which can be uptaken into neurons by MCT2 (Simpson, 1978; Edmond et al., 1987; Auestad et al., 1991; Edmond, 1992). In neurons, ketone bodies are then metabolized in the mitochondria to produce ATP (McKenna, 2012; Brekke et al., 2015). Importantly, the incubation of VMH primary neurons with ketone bodies overrides CD36-mediated FA sensing (Le Foll et al., 2009a) leading us to believe that under HFD condition, ketone bodies sensing is more important than FA sensing (Figure 1B).

Aside from astrocytes, a crucial role for another type of glial cell, tanycytes, has emerged (Chowen et al., 2013; Langlet et al., 2013; Hofmann et al., 2017). These tanycytes line the third ventricle along the hypothalamus and mediate the exchange between the cerebrospinal fluid and adjacent brain areas such as the ARC. Tanycytes send their processes to ARC neurons and astrocytes establishing a “metabolic sensing unit” (Bolborea and Dale, 2013). Using genetically modified mice and fluorescent FA, tanycytes have been shown to preferentially uptake OA vs. PA and OA labeling was found along the tanycytes processes inside the ARC. HFD-feeding altered the uptake of FA in mice with OA and PA showing a similar distribution pattern. Additionally, both were only detected in the tanycyte layer and not in the ARC (Hofmann et al., 2017). Together these studies highlight the important role of tanycytes in uptaking FA in the medio-basal hypothalamus and connecting to different cell type to possibly alter hypothalamic FA sensing. How astrocytes and tanycytes send their information to adjacent neurons and how a specific type/quantity of circulating brain FA can alter this communication and the neuronal activity to control food intake and metabolism remains to be determined.

### How Do Astrocytes Regulate Food Intake During HFD Feeding?

To further understand the role of hypothalamic astrocyte-derived ketone bodies in the regulation of food intake, male Sprague-Dawley rats were trained to eat all of their daily caloric intake on a LFD or 60% HFD for 3 h/day in order to artificially increase their FA levels. Rats fit with a jugular catheter and VMH microdialysis probe were allowed to eat for 24 h on the testing day. Food intake, serum and VMH ketone or FFA levels were assessed over a 6 h period after dark onset (Le Foll et al., 2014). Over the first 3 h, LFD and HFD-fed rats ate the same amount of calories, however, during the second 3 h period after dark onset, HFD-fed rats ate 50% less and consumed fewer meals than LFD-fed rats. As mentioned in the previous paragraph, extracellular VMH FA levels were lower in HFD than LFD rats during the entire 6 h period (Le Foll et al., 2014). Alteration of gene transcription or translation, or the secretion of anorexic gut hormones could explain the underlying mechanism by which this early spike in VMH ketones might induce this delayed decrease food intake. Nevertheless, the pharmacological inhibition of VMH astrocyte ketone production fully restored the intake of HFD to the same levels as LFD during the second 3 h time-period after feeding onset (Le Foll et al., 2014). The intracellular mechanism by which VMH ketone bodies production might alter the activity of VMH neurons responsible for mediating food intake is not fully understood. Using Ca^2+^ imaging to assess the activity of primary VMH neurons, we showed that ketones bodies are able to override CD36-mediated FA sensing in VMH neurons by exciting neurons that were already activated or inhibited by FA (Le Foll et al., 2009a). Thus, we hypothesize that the excitatory effect induced by an excess of ketone bodies provokes an overproduction of ATP or ROS (Paeger et al., 2017) by the neuronal mitochondria that overrides the CD36-mediated FA sensing mechanism, but this hypothesis remains to be tested (Le Foll and Levin, 2016; Figure 1C).

To further understand the effect of ketone bodies on food intake when overweight, ketone bodies levels were measured in DIO and DR rats. Indeed when DIO and DR rats are switched from LFD to HE diet, both become hyperphagic for 3 days (Levin et al., 1997). However, after 3 days on HE diet, DIO rats remain hyperphagic while DR rats lower their intake to their LFD levels (Levin and Dunn-Meynell, 2002; Levin et al., 2003). Even though VMH ketone body levels on the third day of HE diet intake were similar in DIO and DR after dark onset feeding, only DR rats reduced their food intake (Le Foll et al., 2015b). To validate the hypothesis that the reduction of food intake in DR rats on the third day of HE diet intake is due to an increase in VMH ketone bodies level, local astrocyte VMH ketone production was inhibited for 2 h prior to feeding using reverse microdialysis. This inhibition completely reversed their reduced food intake and drastically increased their total daily food intake (Le Foll et al., 2015b), suggesting that DR FA sensing neurons are more sensitive to the overriding effects of ketone bodies on normal CD36-mediated FA sensing than DIO rats. In concert, these results suggest that the hyperphagia observed in HE diet-fed DIO rats might originate in a defective VMH FA and ketone neuronal sensing. In addition, the hyperphagia observed in HE diet-fed DIO rats might also be due to the increase in leptin levels that occurs on the third day of HE diet intake (Levin et al., 2003), resulting in the development of obesity (Park et al., 2011). Finally, given the fact that ketone bodies and CD36-FA sensing control food intake and energy homeostasis through VMH neurons and that these neurons control many physiological processes, we could hypothesize that alteration of neuronal sensing by ketone bodies might alter gut hormone secretion, sympathetic nervous system activity in adipose depots, liver and/or muscle and/or efferent vagal activity to liver and pancreas. Thus, while the short-term inhibitory effects of HFD on feeding appear to be orchestrated by a neuronal-astrocyte coupling, the HFD-induced long-term hyperphagia observed in animals and people may be due additional factors as obesity progressively develops and glial inflammation seems to be one of the main candidate (Figure 1). Indeed, we could hypothesize that HE diet induce astrogliosis in DIO rats but not in DR rats or that DR rats metabolic sensing neurons are more responsive to signals sent by their adjacent astrocytes (Le Foll and Levin, 2016).

### Role of Inflammation

Glial cells constitute around 50% of the total cells in the whole brain (Azevedo et al., 2009; Keller et al., 2018). Glial cells are mainly composed of astrocytes, tanycytes and microglia. As mentioned above, these glial cells create major connections to adjacent neurons and are critical regulators of neuronal activity, especially in the regulation of nutrient uptake and metabolism. The hypothalamic neuronal circuitry, in particular, is highly responsive to obesogenic diets. Indeed, in response to HFD, astrocytes and microglia can proliferate and develop a hypertrophic and reactive phenotype called gliosis (Horvath et al., 2010), which is characterized by the upregulation of specific structural protein such as glial fibrillary acidic protein (GFAP) and ionized calcium binding adaptor molecule 1 (Iba1), respectively (Le Thuc et al., 2017; Avalos et al., 2018). They then produce inflammatory mediators such as TNF-α, IL-1β, IL-6 (De Souza et al., 2005; Garcia-Caceres et al., 2013) and CX3CL1 (only microglia) (Morari et al., 2014). Obese rodents exhibit increased levels of inflammatory cytokines within the hypothalamus (Garcia-Caceres et al., 2013). One possible hypothesis is that this state of low grade inflammation precedes the development of obesity (Le Thuc et al., 2017) and hence plays a causal role, however, the opposite could also be possible. Thaler et al. (2012) has shown that a single day of HFD is sufficient to increase cytokine production in mice whereas others have shown that this requires at least 3 days of HFD intake (Scherer et al., 2012). IKKβ (inhibitor of nuclear factor kappa-B kinase subunit beta) seems to be the major mediator in regulating this glial activation and the subsequent secretion of inflammatory cytokines (Zhang et al., 2008; Douglass et al., 2017). Furthermore, treatment with IL-6 neutralizing antibodies in the hypothalamus improved insulin action in the brain of rodents fed HFD (Sartorius et al., 2012). The inhibition of CX3CL1 in the hypothalamus reduced inflammation, adiposity and glucose intolerance, suggesting that CX3CL1 could mediate the early recruitment of microglia by HFD and thus participate in the induction of the hypothalamic inflammatory response (Morari et al., 2014). Moreover, the combination of high carbohydrate and HFD has been shown to provoke a higher inflammatory response compared to HFD alone (Gao et al., 2017a). Until now, however, it is unknown how HFD-mediated inflammation affects astrocyte-neuronal coupling and modulates hypothalamic FA sensing.

## FA and Reward

In addition to their effect on food intake, FA also exert rewarding properties that vary depending on their type and quantity ingested (Fulton and Alquier, 2018). Reward behaviors can be separated into “liking,” “wanting” and “learning” (Berridge et al., 2009). Reward signals can override homeostatic signals and for example, even satiated after a rich meal, dessert or sweets still sound appealing (Derman and Ferrario, 2018). The mesolimbic system controls reward and feeding behaviors (Cansell and Luquet, 2016) and connects major nuclei involved in reward processes. The VTA neurons project to the NAc as well as to the amygdala and the hippocampus to trigger the release of dopamine (Gardner, 2005). Dopamine is often released in response to motivationally relevant stimuli (Ranaldi, 2014; Holly and Miczek, 2016). Prolonged HFD intake rich in saturated FA decreases this dopamine release in rats and is associated with deficits in the reward system (Schultz, 2002; Stice et al., 2008; Johnson and Kenny, 2010; Michaelides et al., 2012), while a HFD rich in monounsaturated FA such as OA has a protective effect (Hryhorczuk et al., 2018). The lingual application of linoleic acid increased NAc dopamine levels in rats (Adachi et al., 2013) and increased brain c-Fos neuronal activation in the VTA and amygdala (Peterschmitt et al., 2018). Furthermore, excessive intake of saturated fat promotes anxiety and depressive behaviors resulting from neuroinflammatory responses induced by HFD feeding in the NAc (Decarie-Spain et al., 2018). Finally, the depletion of LPL in the NAc increased palatable food preference and food-seeking behavior in chow-diet fed mice injected with TG emulsion in the carotid toward the brain (Cansell et al., 2014). To further assess the role of FA on reward, it would be of interest to study the role of dietary polyunsaturated FA, such as linoleic, EPA or DHA (Hryhorczuk et al., 2016), and determine if these FA induce positive rewarding responses compared to saturated FA. In conclusion, in addition to FA control of food intake, FA exert multiple actions in the brain that all together can regulate food intake and eating behaviors.

## Conclusion and Perspectives

In conclusion, the metabolic sensing unit composed by neurons, astrocytes and tanycytes in the medio-basal hypothalamus is, in part, responsible for the control of food intake and energy homeostasis. Many FA receptors (FAT/CD36, GPCRs) and enzymes (LPL, CPT1) have shown their importance in these metabolic processes. Nevertheless, many questions remain. Do astrocytes send direct signal to neurons through their foot processes? Does inflammation affect neuronal FA sensing and astrocytes ketone bodies production during HFD feeding? Answering these questions would give a better understanding on how the VMH astrocytes and neurons communicate to control food intake and regulate energy homeostasis.

## Author Contributions

The author confirms being the sole contributor of this work and has approved it for publication.

## Conflict of Interest Statement

The author declares that the research was conducted in the absence of any commercial or financial relationships that could be construed as a potential conflict of interest.
